# The significance of structural stigma towards transgender people in health care encounters across Europe: Health care access, gender identity disclosure, and discrimination in health care as a function of national legislation and public attitudes

**DOI:** 10.1186/s12889-023-15856-9

**Published:** 2023-05-31

**Authors:** Felicitas Falck, Richard Bränström

**Affiliations:** 1 Centre for Psychiatry Research, Department of Clinical Neuroscience, Karolinska Institutet, & Stockholm Health Care Services, Region Stockholm, CAP Research Centre, Stockholm, Sweden; 2 ANOVA Clinic, Karolinska University Hospital Stockholm, Norra Stationsgatan 69, 171 76 Stockholm, Sweden; 3 Division of Psychology, Department of Clinical Neuroscience, Karolinska Institutet, Nobels väg 9, 17177 Stockholm, Sweden

**Keywords:** Transgender, Discrimination, Minority stress, Stigma, Policy, Health care seeking

## Abstract

**Background:**

According to the minority stress theory, stigma affects the health of marginalized populations. Previous stigma research has focused on the health effects of individual and interpersonal stigma, paying less attention to structural factors. Laws on legal gender recognition affect the lives of transgender individuals in unique ways. The fact that these laws and population attitudes vary greatly between European countries, offer a unique opportunity to study the role of structural stigma in the lives of transgender individuals. Little is known about how transgender specific structural stigma relates to individual health determinants. Consequently, the aim of this study was to explore the association between structural stigma and access to gender affirming care, gender identity disclosure in health care, and experiences of discrimination in health care across 28 European countries.

**Methods:**

By using multilevel regression, we combined data on health seeking behavior, transgender identity disclosure to health care providers, and experiences of discrimination in health care from 6,771 transgender individuals participating in the 2012 European Union Lesbian, Gay, Bisexual and Transgender survey with a structural stigma measure, consisting of population attitudes towards transgender individuals as well as national legislation on gender recognition. Reasons to refrain from seeking care and discrimination in health care were assessed by categorizing countries as low or high in structural stigma and using Chi-square statistics.

**Results:**

Country-level structural stigma was negatively associated experiences of seeking gender affirming care and positively associated with concealment of being transgender to health care providers. Identity concealment was associated with a lower likelihood of exposure to discrimination in the health care setting across countries regardless of their level of structural stigma. The most prevalent reasons to forgo gender affirming care were shared between low and high structural stigma country groups and centered around fear.

**Conclusion:**

The results highlight the importance of changing stigmatizing legislation and population attitudes to promote access to gender affirming care as well as openness of being transgender towards providers. Measures to decrease discrimination in the health care setting are warranted in high as well as in low structural stigma countries.

## Background

The term transgender refers to individuals whose gender identity or gender expression differ from the sex they were legally assigned at birth [1, 2]. The concept is an umbrella term which encompasses a broad range of individuals with varying identities, gender expressions, experiences, and needs [3]. It includes individuals who identify as men or women as well as those who position their gender identity as non-binary, i.e. between or beyond the categories male and female [4].

The treatment needs of transgender individuals vary and constitute a highly personal matter. Some do not wish to obtain any gender affirming treatment, while others wish to change their primary and/or secondary sex characteristics through hormonal, and/or surgical treatments [5–8]. To be able to live and be perceived in accordance their gender identity, transgender individuals may also wish to change their name, pronoun, and legal gender. Laws on access to gender affirming treatment and change of legal gender vary greatly between countries [9]. In recent years, laws that require transgender individuals to undergo a psychiatric assessment, sterilization, hormonal treatment, or surgery to access legal gender recognition, regardless of the treatment wishes of the individual, have been criticized and amended in some countries whereas they remain in others [10–15]. While the repeal of such legislation is essential from a human rights perspective [16, 17], the impact of legal reform on individual level outcomes of relevance to the health of transgender individuals remains to be scientifically explored.

To align their body with their gender identity, some transgender individuals seek gender affirming treatment such as hormonal treatment or surgery [18–20]. The treatment is effective in alleviating the distress that may arise when the gender identity, body and legal gender of an individual are incongruent, which is commonly referred to as gender dysphoria [8], has been shown to improve psychological wellbeing and quality of life [21], and may improve mental health [22]. The initiation of gender affirming treatment is likely to be dependent on the access and accessibility of healthcare providers who can prescribe such treatments as well as the quality, affordability, and acceptability of their services.

Stigmatization of transgender populations can also affect treatment initiation. Studies indicate that transgender individuals frequently experience stigma and discrimination in the health care setting [17, 23–25], which has a negative impact on their health seeking behavior [26, 27]. However, they also encounter stigma, discrimination, and violence in the wider society [28–30]. Still the impact of societal stigma and legislation on individual level outcomes such as openness to health care providers, health seeking behaviors, and experiences of discrimination during health care encounters remain limited and need to be further explored.

### What is stigma?

Stigma is the result of a process whereby certain groups of people are identified and labelled as different, assigned stereotypical traits, and associated with undesirable characteristics. By creating a distinction between us and them, those who are seen as different are devalued, rejected, excluded, and labeled as deviants, resulting in a loss of status, social, cultural, financial, and political power [31].

Stigma operates at structural, interpersonal and individual levels, as well as across these levels [32]. Individual level stigma involves cognitive, affective, and behavioral responses to discrimination and devaluation, and includes the perceptions that individuals have about themselves as well as their notions of what other people think and feel about them. Interpersonal stigma focuses on interactions between people [33] involving exposure to verbal harassment, physical and sexual violence, unintentional demeaning comments, and a lack of family support [34]. Structural stigma is the most distal form of stigma. It encompasses societal norms, laws, and policies, which restrict the opportunities and resources of stigmatized groups or fail to protect their equal rights [35].

Different stigma levels interact to increase vulnerabilities. For instance, individuals may internalize negative population attitudes about themselves, labelled self-stigma, reducing self-esteem and self-efficacy [36, 37]. They may also attempt to conceal devalued traits to avoid victimization and may become vigilant to rejection [38]. Since an individual is more likely to encounter discrimination upon disclosure of a devalued trait in a high-stigma country [39], concealment may be more effective in reducing exposure discrimination and victimization in such a setting as compared to a low-stigma context. A study on sexual minorities found that concealment was associated with reduced exposure to discrimination and violence in countries with a low as well as a high degree of structural stigma, with higher effects in high-stigma countries [40]. This indicates that structural stigma acts as a moderator on stigma at other levels. It is not known if structural stigma affects experiences of discrimination in the health care setting among transgender individuals.

According to the minority stress model, the stigma and prejudice, which marginalized populations face act as a stressor which drives morbidity and mortality, increasing the risk of mental health problems as well as physical disorders [41–43]. The expectation of being stigmatized may increase blood pressure [44, 45], depression [46], and anxiety [23]. Stigma also has indirect health effects as it restricts access to health protective factors such as financial capital, knowledge, and power [47]. This makes it essential to study if and how the widespread stigma that transgender individuals face is related to their health seeking behavior, openness of being transgender to a health care provider, and experiences of discrimination in the clinical settings as well as reasons for not seeking gender affirming care.

Existing stigma research has primarily focused on individual and interpersonal levels of stigma, with less attention being paid to structural stigma [32]. Research on structural level stigma often measures the attitudes of dominant groups towards stigmatized populations or the contents of stigmatizing policies. However, such stigma measures are rarely combined or linked to individual-level outcomes [35, 48]. A limited number of studies have begun to map the effects of structural stigma on individual-level stigma processes, such as concealment of sexual orientation [40] and disclosure concerns [49]. However, studies on transgender populations largely remain focused on individual and interpersonal stigma, linking them to adverse health outcomes [28, 50], with fewer studies examining the impact of structural risk factors [51–53]. Existing structural-level stigma studies among transgender individuals have explored the effects of US state-level non-discrimination policies on suicidality [54], mood disorders, and self-directed violence [55]. With the exception of a study that looked at gender identity concealment, life satisfaction, and everyday discrimination as a function of structural stigma [56], most studies on stigma towards transgender populations have been conducted in North or South America, highlighting the need to expand research initiatives to other contexts [52]. Furthermore, as studies of transgender populations often sample respondents in health care settings, information on those who refrain from seeking care remains minimal.

The aim of this study was threefold. First, based on the research gaps presented above we wanted to explore how country-level structural stigma, measured as discriminating country-level legislation pertaining to legal gender recognition and population attitudes towards transgender people, is related to healthcare seeking among transgender individuals and to describe if and how reasons to refrain from seeking gender affirming care differ between countries with a high vs. a low degree of structural stigma. Second, we wanted to examine how structural stigma is related to gender identity concealment from health care workers and individual experiences of discrimination in the health care setting. Third, we wanted to understand how experiences of discrimination by a health care provider are affected by gender identity concealment.

Before conducting the statistical analysis we hypothesized that:

1. Transgender individuals living in a country with a high level of structural stigma will report lower likelihood of seeking gender affirming care and a higher probability of seeking such treatment abroad.
2. A higher country-level structural stigma will predict a higher prevalence of gender identity concealment and more frequent experiences of discrimination in health care settings.
3. Individuals who conceal their gender identity to a health provider will experience less discrimination in the health care setting in low- as well as in high-stigma countries but concealment of one´s gender identity will be more effective in preventing exposure to discrimination in high-stigma countries than in low-stigma countries.

## Methods

### Participants

This study relies on data from the European Union Lesbian, Gay, Bisexual, and Transgender (EU-LGBT) survey, which was conducted in 2012 by the European Union Agency for Fundamental Rights [17]. The original aim of the survey was to map discrimination and human rights violations against lesbian, gay, bisexual, and transgender (LGBT) people across the 27 European Union (EU) member states (i.e., Austria, Belgium, Bulgaria, Cyprus, Czech Republic, Denmark, Estonia, Finland, France, Germany, Greece, Hungary, Ireland, Italy, Latvia, Lithuania, Luxembourg, Malta, The Netherlands, Poland, Portugal, Romania, Slovakia, Slovenia, Spain, Sweden, and the United Kingdom) and Croatia. While some results from the survey have been published previously by the European Union Agency for Fundamental Rights (FRA), they have not been analyzed in relation to national legislation and policies for gender minorities. The topics of enquiry included in the survey covered various rights issues with a focus on experiences of discrimination, violence, and harassment. Respondents who indicated that they were transgender received questions on health care issues specific to transgender people.

The questionnaire was developed by a multinational team of LGBT experts and was translated to the 27 languages of the EU member states. Translations were verified by back translation. Cognitive interviews were conducted in five countries to test the validity and relevance of the questionnaire for different subsets of the LGBT population. Participants were recruited online. Invitations to participate in the study were disseminated through local, national, and international LGBT websites. In addition, a Facebook page and a Twitter account were set up to share information about the study. Countries that attracted the fewest responses were targeted with further awareness raising efforts about the study. Respondents completed the survey questionnaire online after having confirmed their consent and understanding of the study purpose. The average time needed to complete the survey was 28 min.

In total 93,079 individuals completed the study. Inclusion criteria in the survey were self-identification as LGBT, being 18 years of age or older, and residing in one of the 27 EU member states or Croatia. As the survey was administered over the internet, internet access was a prerequisite to participate. Only respondents who completed all questions of the survey were included in the data set. Of all survey respondents 6771 individuals (7.3%) defined themselves as transgender. It is the responses of those individuals that are included in this study. For reasons of privacy, details on how study participants defined their gender identity was omitted from the data set before it was shared with us for the purpose of this study. However, from the original survey report we know that 17% of participants defined themselves as trans women, 9% as trans men, 8% as crossdressers, 16% as transgender, 11% as gender variant and 39% as queer/other [17].

### Country-Level characteristics

#### Country-level structural stigma

Based on previous structural stigma research [40, 56–58] we created a continuous measure of structural stigma for each country included in the study. The measure was based on population attitudes towards transgender individuals for each country as well as the national legislation pertaining to legal gender recognition and name change in that particular country in 2012. First, we developed a country-level legislation index, based on information on laws and policies collected by the International Lesbian, Gay, Bisexual, Trans and Intersex Association in Europe [9]. The index of laws was formed by summarizing six items of legislation: (1) lack of legal/administrative procedures for legal gender recognition (4 points), (2) inability to change legal gender on official documents (2 points), (3) inability to change name (1 point), (4) requirement of sterilization to change legal gender (1 point), (5) requirement of medical or surgical interventions to change legal gender (1 point), and (6) requirement of gender identity disorder or medical/psychological opinion (1 point). Each country could be assigned a maximum of 7 points. Certain items were dependent on the existence of others (i.e., a requirement of sterilization, medical/surgical or diagnostic requirement to change legal gender could only exist if a legal or administrative procedure to do so was in place). The index was combined with a measure of population attitudes towards transgender people based on an assessment by the European Commission. In their Eurobarometer survey for 2012, respondents were asked how comfortable they would feel about having a transgender or transsexual person in the highest elected political position in their country, on a scale from 1 to 10 where 1 meant “totally uncomfortable” and 10 meant “totally comfortable” [59]. We combined the standardized law index with the standardized Eurobarometer attitude measure to create our final structural stigma variable. Both variables were coded so that a higher score indicated greater degree of stigma against transgender individuals before the variables were combined. The final structural stigma variable was the averaged mean of the stigma laws variable and public attitudes variable. The score was standardized into z-scores and higher scores indicated higher structural stigma.

In addition to the continuous variable of country-level structural stigma, we categorized all countries based on their score into either low- or high-stigma countries. High-stigma countries included the 14 countries with the highest structural stigma score (i.e., Czech Republic, Greece, Ireland, Italy, Cyrus, Latvia, Lithuania, Malta, Slovenia, Slovakia, Finland, Bulgaria, Croatia, and Romania). Low-stigma countries included the 14 countries with the lowest structural stigma score (i.e., Belgium, Denmark, Germany, Estonia, Spain, France, Luxembourg, Hungary, Netherlands, Austria, Poland, Portugal, Sweden, and the United Kingdom).

#### Country-level income inequality

The Gini coefficient for 2012 was used as a country-level covariate as it has been shown to have a strong association with intolerance and country-level structural stigma in other studies [60].

### Self-report measures

#### Transgender identity

The transgender identity of survey respondents was identified based on their response to the question: “Are/were you a transgender person?” with response options “yes” or “no”. Those who self-identified as transgender were included in the current study. In the original survey participants were also asked to provide more detailed information on their gender identities according to the predefined categories transwoman, transman, female cross dresser, male cross dresser, transgender, gender variant, and queer/other. When requesting permission to use the data from the EU LGBT survey, we were not granted access to individual responses to this question. Consequently, while we know that all study participants regard themselves as transgender, we lack more detailed information on their gender identities.

#### Healthcare seeking behaviors

Readiness to seek health care for being transgender, which will be referred to as gender affirming care throughout this article, in line with current terminology and best practices, was assessed with the question:: “Have you ever sought psychological or medical help for being a trans (transgender) person?”, with the response options: “Yes”, “No”, or “Don’t know. Based on their response, participants were categorized into two groups, those who had sought gender affirming care and those who had not. Respondents who did not know if they had sought care or not (n = 550, 8.1%) were recoded as missing information regarding healthcare seeking to exclude them from further analyses.

Participants who indicated that they had not sought gender affirming care were presented with a list of reasons for refraining to do so, and were asked to select all options pertaining to them. Possible responses were: “I do not want/need help”, “It is not available in the country where you live”, “It is not covered by my country’s public health insurance”, “I cannot afford it due to financial reasons”, “I do not dare to”, “I do not have confidence in the services provided”, “I do not know where to go”, “It takes too much time (including waiting lists)”, “I am afraid of prejudice from the care providers”, “It is too complicated in terms of bureaucracy”, and “I have had previous bad experiences with care providers”. Those who marked the response option “I do not need/want such help” were filtered out in the analysis, leaving only those who had an unmet need for gender affirming care in the statistical calculations.

Readiness to seek gender affirming care abroad was explored through the question: “Have you gone abroad or considered going abroad for medical treatment to alter your physical appearance, including buying hormones over the internet from other countries?”. Participants were presented with the response options: “Yes, I have done”, “Yes, I would do”, “Maybe”, “No, I have not done” and “no, I would not do”. Responses were categorized into two groups, with those who had gone abroad for gender affirming care and those who would do so placed in one group, and those who had not or would not do so in the other group. Those who responded “maybe” were recorded as missing (n = 767, 12.3%). These respondents were removed from the analysis as it was unclear if they had gone or would go abroad to access gender affirming care or not.

#### Gender identity concealment

Gender identity concealment was assessed based on participant responses to the question: “To how many people among the following groups are you open about yourself being transgender?” Survey participants would indicate their degree of openness towards “Medical staff/health care providers” with options being: “none, a few, most, all, does not apply to me”. Concealment was dichotomized into 2 groups, where participants who indicated that they were open to “None” were labeled as concealing, while those who indicated “A few”, “Most” or “All” were considered as open. Those who indicated “does not apply to me” were filtered out/considered as missing (n = 1232, 19.8%) as they could not be categorized as open or not.

#### Discrimination in the health care setting

In the survey participants were asked if they had “accessed health services”. If they indicated “Yes” or “Don’t know” to this question, a follow-up question was posed to assess if they had felt discriminated against on the basis of being transgender when accessing these services during the last year. This follow-up question read: “During the last 12 months, have you personally felt discriminated against because of being (transgender) in any of the following situations?”. This question was followed by a list of situations and groups, one of which was “By health care personnel (e.g. a receptionist, nurse or doctor)”. Responses to this question were used as a basis for statistical analysis regarding experiences of discrimination in the health care setting. The possible answers were “1. Yes, 2. No, and 9. Don’t know”. Those who did not know if they had felt discriminated against or not were recorded as missing (n = 1662, 26.7%) as their answer was unclear.

To examine experiences of discrimination in health care, participants were asked if they had “ever experienced any of the following situations when using or trying to access health care services as a transgender person?”, followed by a list of discriminatory practices. Participants were asked to tick all examples which applied to them. The possible answers were: “Difficulty in gaining access to healthcare. Having to change general practitioners or other specialists due to their negative reaction. Receiving unequal treatment when dealing with medical staff. Foregoing treatment for fear of discrimination or intolerant reactions. Specific needs ignored (not taken into account). Inappropriate curiosity. Pressure or being forced to undergo any medical or psychological test. I have never accessed health care services. None of the above”. Those who marked the response option “I have never accessed health care services” were filtered out in the analysis, leaving only those who had interacted with health care providers in the statistical calculations.

#### Individual-level covariates

Individual-level sociodemographic covariates included age, sex assigned at birth, ethnic minority status, education, annual household income, urbanicity, and relationship status. Participants belonging to an ethnic minority identified themselves as such by ticking the option “ethnic minority (including of migrant background)” after being prompted if they identified as such. Education level was assessed with the question “What is the highest level of education you have achieved?”, response options being “1. No formal education, 2. Primary education, 3. Secondary education, 4. Post-secondary education other than college/university, 5. College/university/higher academic education, and 6. Other”. Annual household income was measured by asking participants to specify if their household’s net combined monthly income was “1. Under lowest quartile, 2. Between lowest quartile and median, 3. Between median and highest quartile or 4. Above highest quartile”, after tax and social insurance fees had been deducted. Urbanicity was measured by asking participants if they currently lived in a“1. City, 2. The suburbs or outskirts of a city, 3. A town, 4. A country village, 5. A farm or home in the countryside.” Participants indicated their relationship status by answering if they were currently: “1. Living together with a partner/spouse, 2. Involved in a relationship without living together, or if they 3. Have no relationship/do not have a partner.”

### Statistical analysis

We used multi-level regression to account for the nested data structure of individuals’ responses within countries. Variables reported on an individual level (i.e., having sought gender affirming care, having sought such care abroad, and concealment about transgender identity in health care settings, as well as experiences of discrimination from health care staff during the last 12 months) were modelled at level 1, while the country-level structural stigma variable was modelled at level 2. Multi-level model estimates are presented as odds ratios with 95% confidence intervals. Only participants with complete answers to all survey questions were included in analyses. In the multi-level analyses, models were adjusted for age, sex assigned at birth, ethnic minority status, education level, annual household income, relationship status, and urbanicity (Level 1) as well as for the Gini coefficient at country level (Level 2). To analyze participants’ responses to questions regarding reasons to not seek health care and different experiences of discrimination, comparisons between countries categorized as low and high structural-stigma countries were conducted using Chi-square calculations. The analyses were conducted using SPSS, version 26.

## Results

### Descriptive statistics

Socio-demographic characteristics of all participants in the EU LGBT Survey 2012 who identified as transgender are presented in Table 1. Most respondents (62%) were assigned male at birth. Younger respondents were more prevalent than older, with 45.9% being 18–29 years old. The sample had a relatively high educational level, with 46.2% indicating having a university education. However 63.3% reported a household income below the median. The vast majority of participants lived in an urban area (86.3%) and most had a partner (52.5%). Structural stigma ranged from − 1.5 for United Kingdom to 2.1 for Lithuania.

**Table 1:** Sociodemographic characteristics of study participants identifying as transgender in the EU LGBT Survey 2012 (n = 6,221)

	n (%)
**Sex assigned at birth**	
Female	2 367 (38.0%)
Male	3 854 (62.0%)
**Age**	
18–29 years	2 809 (45.2%)
30–39 years	1 416 (22.8%)
40–49 years	1 093 (17.6%)
50–59 years	647 (10.4%)
60 years or older	256 (4.1%)
**Ethnic minority status**	
Ethnic minority	435 (7.0%)
**Level of education**	
Less than university	3 350 (53.8%)
University education	2 871 (46.2%)
**Household income**	
Under the lowest quartile	2 391 (38.4%)
Between the lowest quartile and median	1 554 (25.0%)
Between the median and highest quartile	1 226 (19.7%)
Above the highest quartile	1 050 (16.9%)
**Urbanicity**	
Living in an urban area	5 351 (86.0%)
Living in a rural area	870 (14.0%)
**Relationship status**	
Single	2 959 (47.6%)
In a relationship, not living with a partner	1 422 (22.9%)
Live with a partner	1 840 (29.6%)
**Sexual orientation**	
Lesbian	1 111 (17.9%)
Gay	1 443 (23.2%)
Bisexual	1 752 (28.2%)
Heterosexual	894 (14.4%)
Other	736 (11.8%)
Don’t know	285 (4.6%)

### Country variation in healthcare seeking behavior, identity concealment, and experiences of discrimination from health care personnel

To analyze if healthcare seeking behavior, identity concealment, and experiences of discrimination in health varied according to structural stigma levels, we calculated the association between country-level stigma and each of these variables. Results on the association between country-level structural stigma towards transgender individuals and having sought gender affirming care, having sought such care abroad, concealment about one’s transgender identity in health care settings, and experienced discrimination from health care personnel in the last year are presented in Table 2.

**Table 2:** Association between country-level structural stigma and healthcare seeking, gender identity concealment, and discrimination in healthcare

	Multilevel-model estimates		
**Seeking gender affirming care**	**Adj. OR** ^a^	**95% CI**	**Sig.**
County-level structural stigma	0.753	0.571, 0.993	*p* = .045
**Having gone abroad for gender affirming care**	**Adj. OR** ^a^	**95% CI**	**Sig.**
County-level structural stigma	0.786	0.428, 1.419	*p* = .438
**Gender identity concealment in health care settings**	**Adj. OR** ^a^	**95% CI**	**Sig.**
County-level structural stigma	1.286	1.027, 1.611	*p* = .028
**Experience of discrimination by health care personnel during the past 12 months**	**Adj. OR** ^a^	**95% CI**	**Sig.**
County-level structural stigma	0.994	0.835, 1.185	*p* = .950
^a^All models are adjusted for age, sex assigned at birth, ethnicity, level of education, income, relationship status, and urbanicity at Level 1 (i.e., individual level), and for Gini coefficient at Level 2 (i.e., country level), and estimates are presented as odds ratios with 95% confidence intervals.			

### Health care seeking

**Fig. 1:**
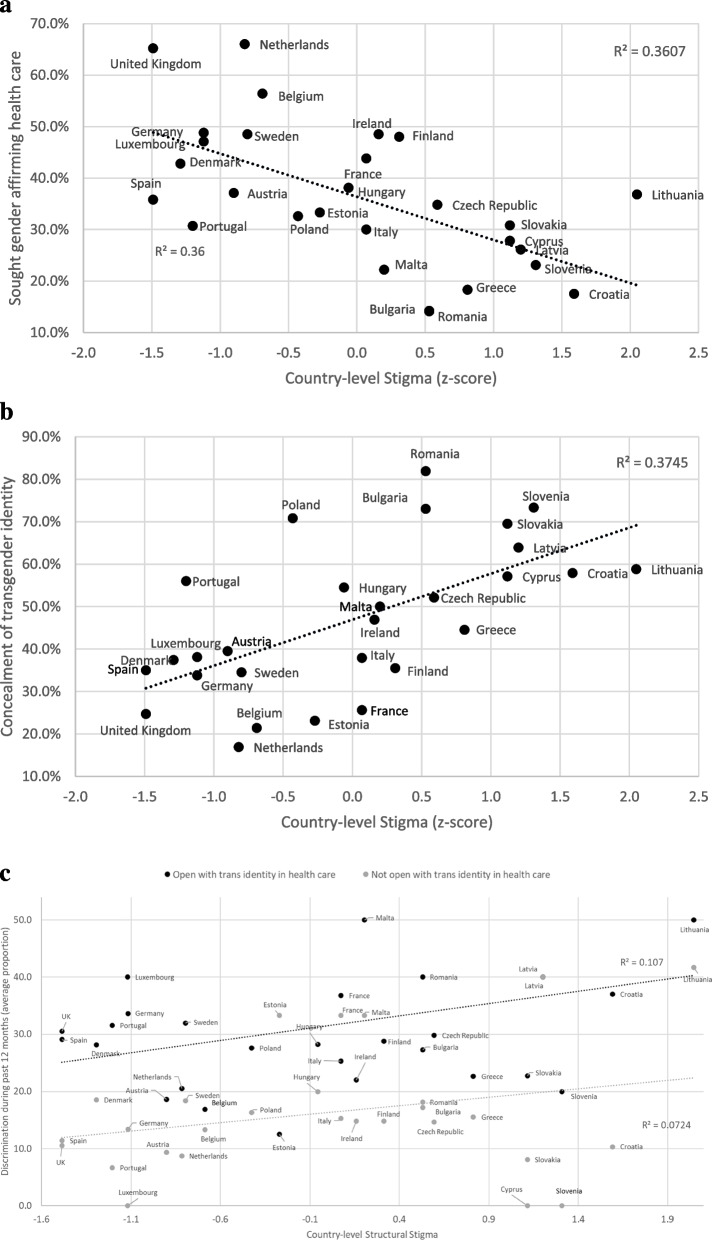
**a **Mean country-level proportion of having sought psychological or medical help for being transgender across Europe by country-level structural stigma. **b **Mean country-level proportion of transgender people reporting concealment of their transgender identity in health care settings, across Europe by country-level structural stigma. **c **Mean country-level proportion of transgender individuals reporting exposure to discrimination during the past 12 months by country-level structural stigma and stratified by openness about transgender identity to health care workers.

Structural stigma was significantly and negatively associated with seeking gender affirming care (Adj OR = 0.753, 95% CI 0.571–0.993, *p* = .045). Essentially individuals living in a high-stigma country were about 25% less likely to seek gender affirming care than those living in a low-stigma country. The mean country-level proportion of transgender individuals reporting having sought gender affirming care by country-level stigma score is presented in Fig. 1a.

Contrary to our hypothesis, we did not find evidence that the likelihood of seeking gender affirming care abroad increases with the level of structural stigma in the country of residence. This association was non-significant (Adj OR = 0.786, 95% CI 0.428–1.428, *p* = .438).

Among those who wanted to seek gender affirming care, but had not done so, reasons for forsaking care were explored for low- and high-stigma countries respectively (Table 3). The most prevalent reasons to forgo such health care were shared between low- and high-structural-stigma country groups. In the low-stigma country group, 41% indicated that they did not dare to seek such services, while 31.9% in high-stigma countries gave this response. The second most prevalent response for low- (33.3%) as well as high-stigma countries (27.2%) was being afraid of prejudice from the care provider. In the low-structural-stigma country group 31.7% of respondents indicated that a lack of knowledge of where to obtain gender affirming care had prevented them from seeking such care, while 26.7% of the respondents in the high-structural-stigma country group did so. Participants in high-stigma countries were significantly more likely to report that unavailability and a lack of national health insurance coverage for gender affirming care prevented them from seeking such care. In contrast time and bureaucracy were more frequent barriers to care in low-stigma countries.

**Table 3:** Reasons for not seeking gender affirming care among those who would like to do so

	Country of residence		
	Low stigma	High stigma	Sig.
It is not available in the country where I live.	2.6%	10.3%	*p* < .001
It is not covered by my country´s public health insurance	9.6%	14.5%	*p* = .005
I cannot afford it due to financial reasons.	20.0%	20.4%	*p* = .838
I do not dare to.	41.0%	31.9%	*p* < .001
I do not have confidence in the services provided.	26.1%	22.1%	*p* = .087
I do not know where to go.	31.7%	26.7%	*p* = .042
It takes too much time (including waiting lists).	16.2%	10.1%	p < .001
I am afraid of prejudice from the care providers.	33.3%	27.2%	*p* = .019
It is too complicated in terms of bureaucracy.	21.1%	14.9%	*p* = .002
I have had previous bad experiences with care providers.	9.9%	8.4%	*p* = .357

### Openness of gender identity to a health care provider

Structural stigma was significantly and positively associated with concealment of one’s gender identity in health care settings (Adj OR = 1.286 95% CI 1.027–1.611, *p* = .028). Individuals living in countries with a high structural stigma were 29% more likely to report concealing their gender identity from health care providers than respondents living in countries with a low structural stigma index. In Lithuania and Croatia, the countries with the highest level of structural stigma, about 60% concealed their gender identity in health care settings whereas 25–35% of participants in the countries with the lowest stigma index, i.e. United Kingdom and Spain, did so. The mean country-level proportion of transgender individuals hiding their gender identity from their health care provider by country-level stigma score is presented in Fig. 1b.

### Experiences of discrimination in health care

Structural stigma was not significantly associated with experiences of discrimination by health care personnel during the last year (Adj OR² =0.994, 95% CI 0.835–1.185, *p* = .95). Instead, reports of discrimination in health care settings were prevalent both in high- and in low-stigma countries.

Across countries and regardless of the level of structural stigma, individuals who concealed their gender identity from health care providers were considerably less likely to report discrimination in health care as compared to those who were open about being transgender *(p <* .001). Among those who concealed their transgender status to their health provider, 68.5% reported that they had not encountered any of the types of discrimination in health care that was outlined in the survey. When looking at those who were open with their transgender identity, the prevalence of transgender-related discrimination in health care almost doubled. Among respondents who were open about being transgender, 36.8% reported no exposure to discrimination in the health setting during the last year.

The mean proportion of trans individuals in each country reporting exposure to discrimination during the past 12 months, by country-level structural stigma, is shown stratified by openness about trans identity to health care workers in Fig. 1c.

Table 4 details the types of discrimination that participants encountered in health care, according to openness and by high vs. low structural stigma country groups. The proportion of individuals who reported no exposure to discrimination in health care while concealing their gender identity was similar for low-stigma countries (69,5%) and high-stigma countries (66,9%). Likewise there was no statistically significant difference in being free from discrimination in health care among those who were open about being transgender to health care providers when comparing low-stigma countries (35,8%) and high-stigma countries (39,7%).

Participants living in high- vs. low-stigma countries who were open about being transgender to their health care provider showed a similar pattern of what types of discrimination that were most commonly exposed to in the health care setting. The most frequent forms of discrimination among these participants were inappropriate curiosity, having their specific needs ignored, and pressure or force to undergo medical or psychological tests. Among those who concealed their gender identity in the health care setting in high- as well as low-stigma countries, foregoing treatment due to a fear of discrimination or intolerant reactions, inappropriate curiosity, and having their specific needs ignored were the most prevalent types of discrimination reported.

**Table 4:** Experiences of transgender-related discrimination in health care according to openness towards health care workers

	All countries			Open in health care			Not open in health care		
	Open in health care	Not open in health care	Sig.	Low-stigma country	High-stigma country	Sig	Low-stigma country	High-stigma country	Sig.
Difficulty in gaining access to healthcare.	22.9%	5.5%	*p* < .001	24.7%	17.3%	*p* < .001	5.0%	6.2%	*p* = .273
Having to change general practitioners or other specialist due to their negative reaction.	22.9%	6.7%	*p* < .001	23.9%	20.1%	*p* = .029	6.7%	6.7%	*p* = .977
Receiving unequal treatment when dealing with medical staff.	18.7%	6.6%	*p* < .001	18.7%	18.6%	*p* = .937	6.6%	6.4%	*p* = .840
Foregoing treatment for fear of discrimination or intolerant reactions.	22.5%	16.4%	*p* < .001	22.4%	23.1%	*p* = .688	16.2%	16.7%	*p* = .803
Specific needs ignored (not taken into account).	31.1%	10.5%	*p* < .001	33.5%	23.9%	*p* < .001	10.9%	9.8%	*p* = .469
Inappropriate curiosity.	35.1%	15.7%	*p* < .001	34.8%	35.8%	*p* = .639	13.3%	19.5%	*p* < .001
Pressure or being forced to undergo any medical or psychological test.	27.6%	7.8%	*p* < .001	29.1%	23.2%	*p* = .002	7.9%	7.6%	*p* = .840
None of the above.	36.8%	68.5%	*p* < .001	35.8%	39.7%	P = .052	69.5%	66.9%	*p* = .271

## Discussion

By combining an unusually large data set on gender identity disclosure, health seeking behaviors, and experiences of discrimination in the health setting among transgender individuals living in 28 countries across Europe with an objective stigma index based on national laws and attitudes, we find evidence that structural stigma predicts whether a transgender individual will come out to their health provider as transgender and seek gender affirming care. The higher the level of structural stigma is in a country, the less likely transgender individuals are to disclose to their healthcare provider that they are transgender and to seek gender affirming care. These findings highlight the important role that general attitudes and laws pertaining to transgender individuals play in shaping individual health outcomes, linking structural and individual stigma levels.

Previous studies have identified individual- and interpersonal-level stigma as deterrents for health care initiation in transgender individuals [26, 27, 61–65]. Several qualitative studies also describe stigmatizing policies and laws as barriers to gender affirming care [65–67]. However, with the exception of a previous study that found an association between state-level structural stigma and the odds of lifetime suicide events in transgender individuals [51], as well as a study which identified lower odds for self-directed violence and mood disorders in transgender individuals living in states that had enacted policies on non-discrimination in employment compared to states that had not [55], the link between structural stigma and health outcomes in transgender populations has remained largely unexplored. To our knowledge no previous study has examined gender identity disclosure to a health care provider and access to gender affirming care as a function of differences in structural stigma across countries. As the first multinational study to document the negative association between transgender specific structural level stigma and the initiation of gender affirming care, this study lends important support to the minority stress theory, which holds that structural conditions, such as laws, policies, and general attitudes towards a minority cause conditions that lead to poor physical [68, 69] and mental health outcomes [43].

Contrary to our hypothesis we did not find evidence that structural stigma was associated with a greater likelihood of seeking gender affirming care abroad. An explanation for this may be that medical and social gender transition is often a visible process. Depending on treatment aims and results as well as the ability to change legal gender on official documents, transitioning may involve a life-long and repeated involuntary coming-out process. Individuals whom others can identify as transgender are more exposed to enacted stigma than those who are regarded by others as cisgender [53], making visual gender conformity important to avoid the scrutiny of others. Existing theories and studies of stigma concealment propose that disclosure of stigmatized traits is dependent on the perceived threat which that openness entails [38, 70–72]. Transgender individuals who live openly in high structural stigma settings are more exposed to everyday discrimination than those living in lower stigma countries, which has a negative effect on their life satisfaction [56]. Against this background it seems plausible that the readiness of an individual to undergo gender affirming care, regardless of whether treatment is prescribed domestically or from abroad, is dependent on what it is like to expose oneself as transgender in the context of where one lives. This may be one of several explanations for why transgender individuals in high-stigma settings were less likely to seek gender affirming care in the country in their country of residence in this study, but also for why they did not compensate for this unmet need of care by seeking such services abroad to a greater extent when living in high-stigma countries as compared to low-stigma countries.

As access to gender affirming care is dependent on the ability of the individual to inform their health care provider that they are transgender, openness is a central aspect of health care for transgender individuals. While previous studies have linked gender identity non-disclosure to health care staff to stigmatization in health care [61, 65], less is known about health care disclosure as a function of structural stigma towards transgender people. Studies on sexual minorities indicate that structural-level stigma is associated with the willingness of individuals to disclose their sexual orientation to health care providers [73–75]. Sexual minority men living in countries with a higher level of structural stigma, measured as national laws, policies, and general attitudes towards sexual minorities, had lower odds of disclosing their sexual orientation to providers when being tested for HIV as compared to those living in lower-stigma countries, suggesting that structural stigma may affect openness to health care providers [76]. The results of this study expand existing research that links structural factors and identity concealment in health care to include transgender individuals. As such it contributes to filling an important knowledge gap.

While structural stigma was negatively associated with the likelihood of being open to providers as transgender, it was not statistically associated with experiences of discrimination in the hands of health care providers. Instead discrimination from providers was rampant across countries. While it is possible that structural stigma towards transgender individuals may be truly unrelated to how health care providers treat their patients, this appears unlikely. A previous study, although not on transgender individuals, shows that the attitudes of medical practitioners are similar to those of their countrymen [77]. The fact that we did not find a statistically significant association between structural stigma and experiences of discrimination at the hands of health care providers may be caused by some of the weaknesses of this study. The data on enacted discrimination in health care relied on reports of subjective experiences of discrimination, rather than objective measures of unfair treatment. We do not know if participants living in a low-structural-stigma country were objectively exposed to discrimination with equal frequency or severity as those living in a country with a high level of structural stigma or not, or if their recollection of such events is different. Internalized stigma has been associated with a low self-esteem and a greater acceptance of stereotyped attitudes in individuals with mental health problems [78]. It may well be that transgender individuals who live in countries where structural stigma is rampant and justified by others fail to identify when they are exposed to differential treatment, leading to them to underreport such instances. Similarly those who live in a setting where stigma towards transgender individuals is considered inappropriate may be more prone to identify and remember such instances as acts of discrimination. As such, the finding that structural stigma does not predict a higher risk of stigma exposure in the health setting, may be influenced by the scope of the survey questions as well as recall bias.

Moreso, as participants living in high-stigma countries were open to health care providers about being transgender to a lesser extent than those living in lower-stigma settings, they represent a more selected group. Previous research indicates that socioeconomic, ethnic, and other factors may influence transgender individuals’ exposure to bias [25, 79] and consequently their willingness to come out to a health care professional as transgender [80]. We do not know if the participants who chose to come out in high-stigma nations were privileged in ways that compensate for the stigma that their gender identity entailed, and whether this affects the study results. Moreso, transgender individuals in high-stigma countries could be more selective in choosing which health care professionals they come out to, leading them to experience less stigma than if they were to come out more broadly. This could unfortunately not be examined more closely in the present study, as it was limited by the questions that were posed in the EU LGBT survey.

The fact that fear of prejudice from health care providers was the second most prevalent reason to refrain from seeking health care for being transgender in high- as well as in low-stigma countries, highlights the importance of understanding the root causes of interpersonal stigma in health care. Although this study did not find an association between structural stigma and experiences of discrimination from providers, such a link cannot be ruled out. Instead, the potential association between structural stigma and interpersonal stigma from health care providers should be further explored in future studies.

While discrimination in the health setting was prevalent across countries, concealment of being transgender was associated with a substantially reduced risk of encountering discrimination in the health care settings in low- as well as high-structural-stigma nations. Individuals who were open to health care providers were twice as likely to report exposure to discrimination as compared to those who did not disclose their transgender status. This finding adds to and follows previous studies that indicate that concealment can protect transgender individuals against victimization [38] and everyday discrimination [56]. Although non-disclosure of a stigmatized trait can be a functional coping strategy, it has been associated with negative health outcomes [81] such as depressive symptoms, [82], anxiety, a negative mood, and poor self-esteem [83] as well as increased psychological strain [84]. Individuals who fail to tell their provider that they are transgender may miss out on sex specific screenings [81], such as cervical pap smears and screening for prostate cancer as well as referrals to gender affirmative health care services. As the desire to live and be accepted by others in line with one’s gender identity is an inherent feature and a diagnostic criteria of gender dysphoria [85] and failure to do so is associated with a lower life satisfaction in transgender populations [56], being able to come out to a health care professional without the fear of stigma is also important in its own right. Consequently, while the results of this study indicate that legal reform is important to ensure openness and health care access for transgender individuals, active measures to combat stigma of transgender individuals in health care remain warranted in low- as well as in high-structural-stigma settings.

A limitation related to the data of this study is that information on how participants defined their gender identity was deleted from the data set before it was shared with us. While all participants identified as transgender, we have not been able examine if there are any differences concerning openness, health care initiation or discrimination between different identity categories. Similarly the number of participants from some countries was too low to enable us to analyze potential differences between those assigned male vs. female at birth. The ways in which structural stigma might affect different subsets of transgender individuals, such as those who define themselves as men/women as opposed to those how have a non-binary identity, would be interesting to examine in future studies. It would also be interesting to explore if and how stigma can contribute to the explanation of why transgender individuals assigned female at birth now seek gender affirming care in increasing numbers as compared to those assigned male [86–88].

While the survey that this study is based upon was conducted 12 years ago, it appears likely that the association between structural stigma and factors of relevance for the health and wellbeing of transgender individuals, such as openness and access to gender affirming care, still holds. Although the legislation on gender recognition has remained intact or changed only slightly in several EU countries since then, other countries have made substantial changes to their legislation, making legal gender recognition a less restrictive, pathologizing and authoritarian process. Belgium, Denmark, Iceland, Ireland. Luxembourg, Malta, Norway, and Portugal now treat legal gender recognition as a matter of self-determination and other countries such as Sweden have removed mandatory sterilization as a requirement to change legal gender [89, 90]. Parallel to these developments, the number of individuals who seek gender affirming care has increased in several countries [91, 92], sparking a debate on the cause behind this shift. While the results of this study would suggest that transgender individuals with unmet treatment needs may become more prone to seek gender affirming care as structural stigma decreases, this remains to be explored in future studies. The recent changes in laws regarding legal gender recognition in the EU provide an excellent opportunity to investigate this further.

## Conclusion

This study provides important new insights into the association between structural stigma and individual-level outcomes such as openness to a health care provider and health seeking behaviors among transgender individuals. While the study was limited by the questions posed in the EU LGBT survey, it benefits from an extensive data set which is unusual to see in research on transgender individuals. Another strength is its reliance on objective data regarding national legislation and population attitudes towards transgender individuals.

The results of the study point to the importance of transgender-friendly legislation and initiatives to affect the attitudes of wider populations towards transgender individuals, in order to meet the health care needs of this varied and marginalized population. Interventions to decrease stigma towards transgender individuals who come out to their health care provider are warranted in high- as well as lower-structural-stigma settings. Since transgender people face specific structural challenges, such as barriers to legal gender recognition and gender affirming care that meet their needs, it is essential to continue to explore how this affects their health as well as health-related needs.

## Data Availability

The data that support the findings of this study are available from the European Union Agency for Fundamental Rights (https://fra.europa.eu/en/about-fra), but restrictions apply to the availability of these data, which were used under license for the current study, and so are not publicly available. Data are however available from the authors upon reasonable request and with permission of the European Union Agency for Fundamental Rights.

## Abbreviations

EU: European Union
EU: LGBT Survey - European Union Lesbian, Gay, Bisexual and Transgender survey
FRA: European Union Agency for Fundamental Rights
LGBT: Lesbian, Gay, Bisexual, Transgender
